# Lung Microbiome Differentially Impacts Survival of Patients with Non-Small Cell Lung Cancer Depending on Tumor Stroma Phenotype

**DOI:** 10.3390/biomedicines8090349

**Published:** 2020-09-13

**Authors:** Olga Kovaleva, Polina Podlesnaya, Madina Rashidova, Daria Samoilova, Anatoly Petrenko, Irina Zborovskaya, Valeria Mochalnikova, Vladimir Kataev, Yuri Khlopko, Andrey Plotnikov, Alexei Gratchev

**Affiliations:** 1 N.N. Blokhin National Medical Research Center of Oncology, 115478 Moscow, Russia; ovkovaleva@gmail.com (O.K.); polina.pod@yandex.ru (P.P.); madina.211@mail.ru (M.R.); dashasam@mail.ru (D.S.); epigenome@inbox.ru (A.P.); klopik@list.ru (I.Z.); mochalnikova70@yandex.ru (V.M.); 2 Institute for Cellular and Intracellular Symbiosis, The Ural Branch of the Russian Academy of Sciences, 460000 Orenburg, Russia; vladimir0334@yandex.ru (V.K.); 140374@mail.ru (Y.K.); protoz@mail.ru (A.P.)

**Keywords:** non-small cell lung cancer, prognosis, iNOS, microbiome, macrophage, stroma, Treg

## Abstract

The link between a lung tumor and the lung microbiome is a largely unexplored issue. To investigate the relationship between a lung microbiome and the phenotype of an inflammatory stromal infiltrate, we studied a cohort of 89 patients with non-small cell lung cancer. The microbiome was analyzed in tumor and adjacent normal tissue by 16S rRNA amplicon sequencing. Characterization of the tumor stroma was done using immunohistochemistry. We demonstrated that the bacterial load was higher in adjacent normal tissue than in a tumor (*p* = 0.0325) with similar patterns of taxonomic structure and alpha diversity. Lung adenocarcinomas did not differ in their alpha diversity from squamous cell carcinomas, although the content of Gram-positive bacteria increased significantly in the adenocarcinoma group (*p* = 0.0419). An analysis of an inflammatory infiltrate of tumor stroma showed a correlation of CD68, iNOS and FOXP3 with a histological type of tumor. For the first time we showed that high bacterial load in the tumor combined with increased iNOS expression is a favorable prognostic factor (HR = 0.1824; *p* = 0.0123), while high bacterial load combined with the increased number of FOXP3+ cells is a marker of poor prognosis (HR = 4.651; *p* = 0.0116). Thus, we established that bacterial load of the tumor has an opposite prognostic value depending on the status of local antitumor immunity.

## 1. Introduction

Non-small cell lung cancer (NSCLC) is one of the most common and difficult to treat cancers in the world. In Russia today, lung cancer ranks first in morbidity and mortality among all types of cancer. Despite the fact that these diseases are well studied, reliable prognostic requirements of this pathology are not convincing enough. One of the new approaches to predict the course and possibly effectiveness of immunotherapy (which is actively used in the treatment of NSCLC) may be a comprehensive analysis of the unique phenotypic characteristics of the tumor stroma, namely the cellular composition of the inflammatory infiltrate together with the composition of its microbiome.

Although the microbiota consists of viruses, bacteria, archaea, protists and fungi, predominant microorganisms in the microbiota of the human respiratory tract are bacteria. The lung had long been considered a sterile organ until Hilty M et al. identified lung resident microbiomes in healthy individuals using metagenomic sequencing [1]. The human microbiota is primarily colonized by Firmicutes, Bacteroidetes, Proteobacteria, Actinobacteria and Fusobacteria [2]. Normally, the main representatives of the lower respiratory tract microbiome are the genera *Pseudomonas, Streptococcus, Fusobacterium, Megasphaera* and *Sphingomonas* [1,3,4]. Recently, correlations of the lung microbiome with various chronic diseases (asthma, COPD, etc.), as well as lung cancer [5] have been described. Very few studies have investigated the association between the lung microbiome and clinicopathological features of lung cancer. It has been suggested that different patterns of the lung microbiome are associated with histological types and stages of lung cancer [6]. K. Leigh Greathouse et al. have analyzed 143 lung cancer samples and found that genera *Acidovorax, Klebsiella, Rhodoferax* and *Anaerococcus* were enriched in lung squamous cell carcinomas compared to lung adenocarcinomas [7]. Yan et al. have showed that an increased abundance of *Capnocytophaga*, *Selenomonas*, *Veillonella* and *Neisseria* is typical for both squamous cell lung cancer and adenocarcinoma, and those bacteria can potentially be used as a marker of lung cancer [8]. Few studies showed a link between lung bacteria and distant metastasis of lung cancer [9].

In contrast to the microbiome, the cells’ phenotype of the stromal component of lung tumors and its prognostic significance have been studied quite well. A large number of alternatively activated M2 macrophages are traditionally considered a marker of poor prognosis [10], however, many modern studies also show good prognostic significance for both M1 and M2 macrophages [11]. In many studies the presence of a large number of CD3+ and CD8+ cells has been associated with a favorable prognosis and improved survival, while the presence of a large number of Treg (FOXP3+) has been often associated with a worse prognosis [12].

In the tumor microenvironment, the integration of anti-inflammatory signals from tumor cells and proinflammatory signals from bacteria can occur. How this integration occurs, how the tumor’s microbiota is formed and changed, which phenotype the stroma cells acquire and how this affects the course of the disease, is currently unknown. The relationship of the microbiome, tumor stroma immunoreactivity, and clinical outcome has been described in a single study on pancreatic adenocarcinoma [13].

In this study we aimed to analyze the composition of the microbiome in NSCLC tumors depending on their clinical and morphological characteristics and the phenotype of an inflammatory infiltrate of tumor stroma, as well as prognostic significance of the microbiome.

## 2. Materials and Methods

### 2.1. Ethics Statement

The samples were collected in accordance with the guidelines issued by the Ethics Committee of the N.N. Blokhin National Medical Research Center of Oncology. All patients gave written informed consent (available upon request). The study was performed in accordance with the principles outlined in the Declaration of Helsinki.

### 2.2. Sample Collection

Tumor tissues and matched histologically normal adjacent tissues were obtained from patients after surgical resection and were stored in liquid nitrogen. Diagnoses were verified by histopathology, and only samples containing 70–80% or more tumor cells were used in the studies. Matched controls were histologically confirmed to be normal epithelial cells. The tumor samples were characterized based on the tumor-node-metastasis according to the International System of Classification of Tumors, according to the staging classification of the Union for International Cancer Control (UICC, version 2009) [14], and using the criteria for classification developed by the World Health Organization (WHO) [15]. The NSCLC group included 44 (49%) adenocarcinomas and 45 (51%) squamous cell carcinomas. The mean follow-up for living patients was 33 months (range, 3–104 months). Overall survival (OS) was defined as the interval between surgery and death or between surgery and the last follow-up for surviving patients. Among the 68 patients who were recruited, 31 (46.0%) died and 37 (54.0%) remained alive during the follow-up period. Other specimens’ characteristics are presented in Table 1.

### 2.3. Immunohistochemical Study

Formalin-fixed, paraffin-embedded NSCLC tissue samples were step-sectioned and deparaffinized using the standard protocol. Endogenous peroxidase activity was blocked with 3% hydrogen peroxide for 10 min. HIER was provided in Tris-EDTA (pH 9.0) in a Decloaking Chamber (Biocare Medical, Concord, CA, USA). Sections were incubated with primary antibodies at room temperature: anti-CD206 (Sigma-Aldrich, St. Louis, MO, USA HPA004114), anti-CD68 (Genemed, South San Francisco, CA, USA 61-0184), anti-CD163 (Clone 10D6; BIOCARE, USA), anti-iNOS (Sigma, St. Louis, MO, USA SAB5500152), anti-CD8 (Genemed, South San Francisco, CA, USA 61-0124), anti-CD3 (Genemed, South San Francisco, CA, USA 61-0011) and anti-FOXP3 (Cell Signaling, Denvers, MA, USA #98377). A PowerStain 1.0 PolyHRP DAB kit (Genemed, South San Francisco, CA, USA 54-0017) was used for the detection. Afterwards counterstaining with hematoxylin was performed.

To score the immunostaining results for macrophages (CD68, CD163 and CD206) and T-cells (CD3 and CD8), we randomly selected five representative high-power microscopic fields (×400 magnification) of the tumor sample per section, counted the numbers of positively stained cells, and photographed the sections with a digital camera (Olympus BX53F, Tokyo, Japan). Necrotic areas were ignored. The mean percentages of stained cells were counted as 0 (negative), 1 (≤10%), 2 (11–50%) and 3 (>50%). Foxp3 expressions were evaluated according to the average number of positively stained cells in 5 randomly and averagely selected 400 × high-power fields (HPF) in each case: 0 (no positive cells), 1 (1–5 positive cells), 2 (6–25 positive cells) and 3 (>25 positive cells) per HPF. Samples with scores 0–1 for CD206, CD8 and FoxP3 were combined in a group with low expression and samples with scores 2–3 were combined in a group with high expression. For CD68, CD163 and CD3 samples with scores 0, 1 and 2 were combined in a group with low expression and samples with a score of 3 represented a group with high expression [16].

For iNOS immunohistochemical staining was scored in tumor cells. Tumor staining was classified as positive when clear cytoplasmic staining was present in ≥1% of tumor cells. Since there are no clinically accepted thresholds for iNOS expression, the following cutoff was used for this stain expression: low 1–10% and high >10% of the tumor cells showing cytoplasmic positivity (Figures S1 and S2).

### 2.4. Quantitative PCR (qPCR)

Quantitative real-time PCR was performed to assess the abundance of the 16S gene present in a subset of normal and tumor tissue pairs. The following primers were used: F3106 (5′-CCTACGGGNGGCWGCAG-3′) as the forward primer and R3106 (5′-GACTACHVGGGTATCTAATCC-3′) as the reverse primer [17]. The PCR program was as follows: 95 °C for 5 min, 40 cycles of 95 °C for 15 s, 55 °C for 30 s and 72 °C for 1 min. A total of 100 ng of extracted DNA and 0.5 μL of each primer (10 pmol) were added to 4 μL of the PCR mix—qPCRmix-HS-SYBR (Evrogen, Moscow, Russia), and DNA-free water was added up to 20 μL of the total volume. All reactions were performed in triplicates. A negative control containing DNA-free water instead of DNA was used for each PCR run. The real-time qPCR data analysis was performed with the BioRad software (Bio-Rad CFX Manager 3.1, Hercules, CA, USA) with a manually set threshold. For the purposes of analysis, the metric was a number of cycles to the cross threshold (Ct value) as a measure of *16s* rRNA gene load and hence bacterial burden. A higher bacterial load resulted in a lower number of cycles to the cross threshold, that is, a lower Ct value [18].

### 2.5. 16S rRNA Gene Library Preparation and MiSeq Sequencing

DNA extraction from tissues was performed using a DNA FFPE kit (Qiagen, Hilden, Germany) according to the manufacturer’s instructions for capturing bacterial DNA. The quality of the extracted DNA was assessed with electrophoresis in 1% agarose gel and a Nanodrop 8000 (Thermo Fisher Scientific, Waltam, MA, USA). The DNA concentration was quantified using a Qubit 4.0 Fluorometer (Thermo Fisher Scientific, Waltam, MA, USA) with a dsDNA High Sensitivity Assay Kit (Thermo Fisher Scientific, Waltam, MA, USA).

Preparation of the DNA libraries was performed according to the Illumina protocol (Part #15044223, Rev. B.) with primers targeting the V3–V4 regions of the SSU ribosomal RNA (rRNA) gene, F3106 as the forward primer and R3106 as the reverse primer [17]. The reaction mixture (10 µL) contained both primers, 0.1 µM each; 80 µM dNTPs and 0.2 U Q5 High-Fidelity DNA Polymerase. The following PCR program was used: 95 °C for 3 min, 40 cycles 95 °C for 30 s, 56 °C for 30 s, 72 °C for 30 s and final extension 72 °C for 5 min. For each reaction three replicates were amplified. Then the replicates were mixed together, and cleaned up using Agencourt AMPure XP beads (Beckman Coulter, Brea, CA, USA). Paired-end 2 × 300 bp sequencing was performed on the MiSeq platform (Illumina, San Diego, CA, USA) with the Reagent Kit v.3 (Illumina, San Diego, CA, USA).

DNA libraries preparing, sequencing and bioinformatics treatment were performed in the Center of Shared Scientific Equipment “Persistence of microorganisms” of Institute for Cellular and Intracellular Symbiosis UrB RAS, Orenburg, Russia.

### 2.6. Bioinformatics Treatment

At the first stage, the raw reads obtained as a result of sequencing were evaluated with FastQC v. 0.11.7. Evaluation was necessary to determine the parameters of further processing, and included an assessment of quality and length of reads, the presence of adapter sequences. Paired-end reads were merged with a minimum overlap of 40 bp and a *p*-value of 0.0001 using PEAR v. 0.9.10 (http://www.exelixis-lab.org/web/software/pear) [19]. Adapter sequences were removed with Trimmomatic v 0.36 (http://www.usadellab.org/cms/?page=trimmomatic) [20]. After merging and adapters removal, the reads were re-evaluated with FastQC v. 0.11.7. Subsequent treatment of merged reads was conducted with Usearch v. 9.2.64 (http://drive5.com/usearch) [21] and included quality filtering (expected error or maxee less than 1.00) and amplicon size selection (420 bp minimal size). Evaluation of the filtering quality was carried out with FastQC v 0.11.7. The next stage included dereplication and clustering of the filtered reads. As a result of dereplication and clustering, operational taxonomic units (OTUs) were formed. Chimeric sequences were detected and removed using the UCHIME2 algorithm [22]. Final OTUs were aligned to the initial merged reads using global alignment (usearch_global tool) at a 97% level of similarity. As a result of global alignment, the number of merged reads corresponded to every OTU was estimated. Contaminant OTUs were identified and removed via the usearch_ublast command by matching the sequences of trial samples and negative control samples. The taxonomic classification of sequences was conducted using the RDP reference database (http://rdp.cme.msu.edu/index.jsp) [23]. For OTUs with a taxonomic position estimated at a low level of support (ab_score less than 0.7), taxonomy was determined using the NCBI database https://blast.ncbi.nlm.nih.gov. OTUs identified as a host (human) were removed from the dataset.

#### Availability of Data

Raw sequence data and metadata are available at the NCBI Sequence Read Archive under accession numbers SRR12264494-12264543, BioProject PRJNA647170 and BioSamples SAMN15577976-15578025.

### 2.7. Statistical Analyses

Diversity of microbiomes within samples (alpha diversity) was evaluated with indices Chao1, ACE, inverse Simpson and Shannon. The similarity of microbiomes between samples (beta diversity) was assessed using the Bray–Curtis distance. To visualize the similarity of microbiomes between samples, a principal coordinates analysis (PCoA) was performed. Taxa that were significantly different between NSCLC and normal tissues we identified with a MicrobiomeAnalyst [24], developed for microbiome statistics applications. Differences in the overall microbial composition between NSCLC and adjacent normal tissues and other groups were assessed by a Wilcoxon rank-sum or Mann–Whitney nonparametric test.

Immunohistochemistry (IHC) statistical analysis was performed using GraphPad Prism ver. 8.3 by GraphPad Software (San Diego, CA, USA). χ^2^ and Fisher exact tests (for categorical variables) were used to compare the differences between the expression of CD68 and other markers and clinicopathological parameters of NSCLCs. Continuous variables were compared between groups by a Wilcoxon rank-sum or Mann-Whitney nonparametric test. Survival length was determined as a time period from the date of surgery to the date of death or the last clinical attendance. Survival curves were derived using the Kaplan–Meier method, and differences between curves were analyzed using the log-rank test. In all analyses, *p* values ≤ 0.05 were considered statistically significant.

## 3. Results

### 3.1. Clinical Samples

This study included 89 patients operated for NSCLC at the N.N. Blokhin National Medical Research Center of Oncology. All samples were paired, that is, they consisted of histologically verified tumor tissue and a sample of conditionally normal lung tissue of the same patient located as far as possible from the tumor. In the study we took samples of two main histological types: adenocarcinoma and squamous lung cancer. Other histological types of malignant lung tumors were not included in the study. Clinical characteristics of the 89 patients are presented in Table 1.

### 3.2. Characterization of Lung Bacterial Communities

To analyze the composition of the microbial community, the 16S rRNA gene was sequenced in 26 pairs of DNA samples from NSCLC tumor and corresponding adjacent tissue samples. The sequenced samples included 14 adenocarcinomas and 12 squamous cell carcinomas. In total, 12 samples belonged to the I–II stages of the disease and 14 samples to the III–IV stages, 9 samples were from patients without regional metastases and 14 samples were of high and moderate differentiation.

**Figure 1:**
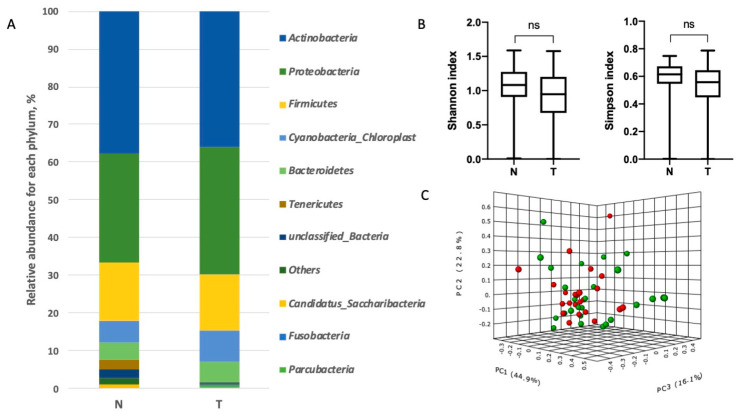
Taxonomic composition of the microbial community of lung tissues on the level of phylum. (**A**). Characterization of non-small cell lung cancer (NSCLC) microbiota. Relative abundance at the phylum level for tumor (T) and normal tissue (N) samples. (**B**). Taxonomic α-diversity calculated with the Shannon and Simpson indices between N and T groups (ns = non significant). (**C**). Principal coordinates analysis (PCoA) plot based on the Bray–Curtis distances of NSCLC microbiomes between tumor and normal tissues (PERMANOVA) F-value: 0.81483; R-squared: 0.016692; *p*-value < 0.507 (green ball–tumor, red ball–normal tissue).

**Figure 2:**
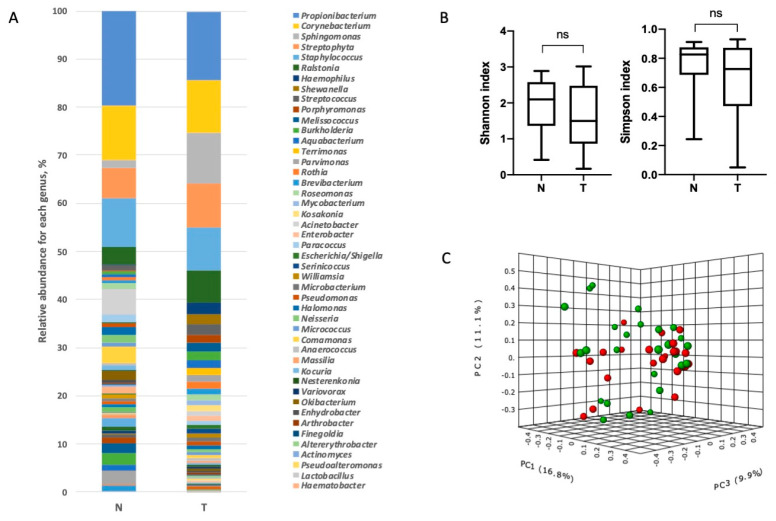
Taxonomic composition of the microbial community of lung tissues on the level of genera. (**A**). Characterization of NSCLC microbiota. Relative abundance at the genus level for tumor and normal tissue samples. (**B**). Taxonomic α-diversity calculated with the Shannon index and Simpson indices between N and T groups (ns - non significant). (**C**). PCoA plot based on the Bray–Curtis distance of the NSCLC microbiome between tumor and normal tissues (PERMANOVA) F-value: 1.2264; R-squared: 0.024913; *p*-value < 0.204 (green ball–tumor, red ball–normal tissue).

Analysis of the microbiome taxonomic composition in the lung tissue samples revealed the presence of 10 phyla (Figure 1 and Table S1) and 280 genera (Figure 2 and Table S2). Among the top phyla by relative abundance we found *Firmicutes, Bacteroidetes, Proteobacteria, Actinobacteria* and *Fusobacteria*, which have been described previously [2]. There were no significant differences in the relative abundance of the microorganisms at the phylum level between tumor and adjacent normal tissues (Figure 1).

Next, we analyzed the relative abundances of bacteria at the phylum and genus levels in the tumor and adjacent normal tissue. *Actinobacteria, Proteobacteria, Firmicutes*, and *Bacteroidetes* were the predominant phyla of microorganisms found both in tumor and adjacent tissue samples (Figure 1). For the analysis, bacterial genera with an abundance level of more than 0.1% were taken into account. There were 70 such dominant genera.

No significant differences for taxonomic alpha diversity were observed between tumor and normal adjacent tissue (Shannon and Simpson indices). To evaluate the similarities between all samples, distances, calculated on the basis of the unweighted UniFrac metrics, were visualized by a PCoA plot. There was no significant distinct separation between the tumor and normal adjacent tissue groups at the levels of both the phylum and genera (Figure 1 and Figure 2).

Next, the taxonomic composition of the microbial communities was compared at the genus level in lung tumors of various histological types, stages and grades. For this analysis we selected 40 genera, each of them comprised of more than 0.5% of the total abundance. We also conducted an analysis of alpha diversity in each group at the genus level using Shannon and Simpson indices, which takes into account both the number of taxa and relative abundance of every taxon in a sample. The analysis showed a statistically significant difference in the relative abundance of three genera *Acinetobacter, Halomonas* and *Chryseobacterium*. We found decrease of the percentage of those bacteria in the tumors, compared with adjacent normal tissue samples (Table S2).

**Figure 3:**
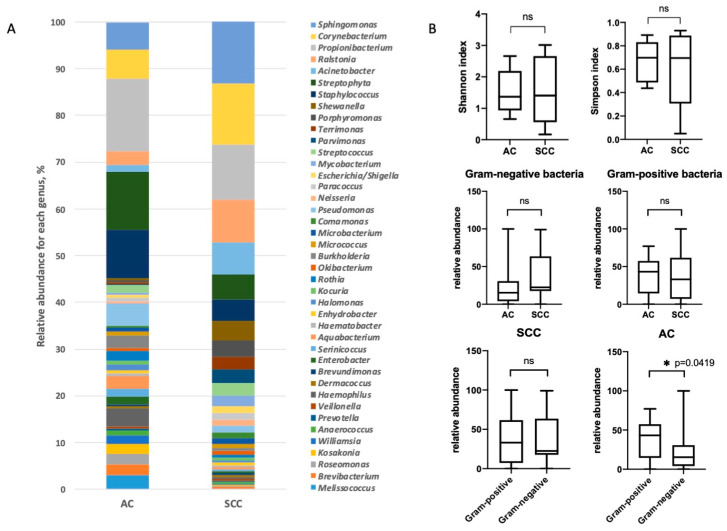
Taxonomic composition of the microbial communities at the genus level in lung tumors of different histological types. (**A**). Relative abundances of the dominant bacterial genera in the microbiota of adenocarcinoma (AC) and squamous cell carcinoma (SCC) groups (**B**). The alpha diversity was calculated with Shannon and Simpson indices for the AC and SCC groups. The percentage of Gram-positive and Gram-negative bacteria in groups of different histological types is shown (∗-statistically significant).

The analysis of the relative abundance of 40 dominant bacterial genera in the groups of adenocarcinomas and squamous cell carcinomas did not reveal any differences (Figure 3A and Table S3). The alpha diversity of microbial communities at the genus level in tumors of different histological types also did not differ. However, it is interesting to note that in the adenocarcinoma group Gram-positive bacteria prevailed significantly (*p* value = 0.0419) over Gram-negative bacteria. For the squamous cell carcinoma group, such a difference was not found. The relative abundance of Gram-positive and Gram-negative bacteria did not differ between the adenocarcinoma and squamous cell carcinoma groups (Figure 3B).

**Figure 4:**
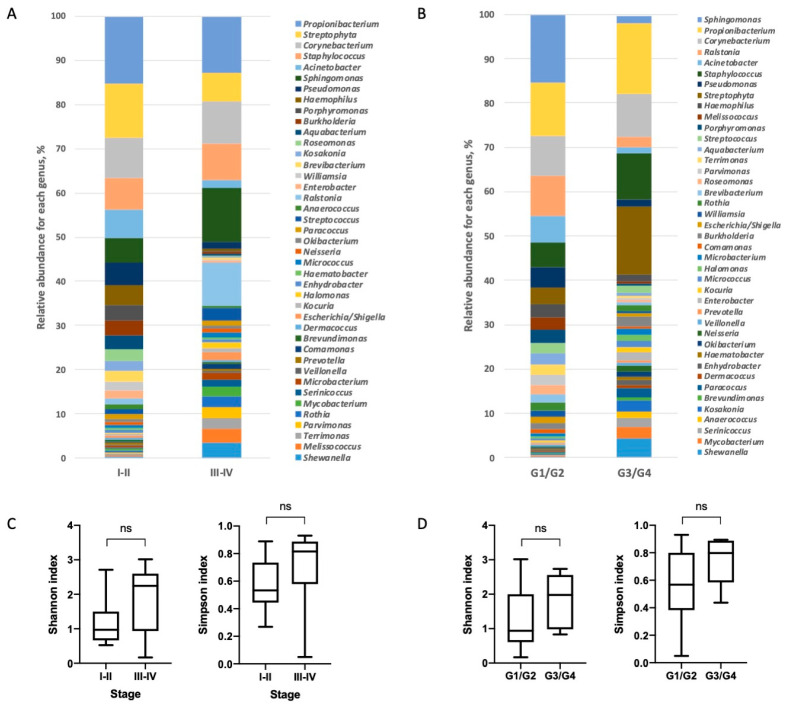
Taxonomic composition of the lung tumor microbiomes at the genus level for tumors at different stages (**A**) and with various differentiations (**B**). Taxonomic alpha-diversity calculated with Shannon and Simpson indices between the early and late stages (**C**) and low and high grades (**D**).

The taxonomic analysis of the microbiome composition of NSCLC tumors at different stages showed significant differences between 11 bacterial genera by their relative abundances: *Corynebacterium, Sphingomonas, Pseudomonas, Burkholderia, Aquabacterium, Streptococcus, Neisseria, Halomonas, Parvimonas, Rothia* and *Kocuria*. It is worthy to note that the percentage of genera *Pseudomonas, Burkholderia* and *Aquabacterium* was lower at the late stages compared to the early stages, while the genera *Corynebacterium, Sphingomonas, Streptococcus, Neisseria, Halomonas, Parvimonas, Rothia* and *Kocuria* demonstrated the opposite pattern of differential distribution (Figure 4 and Table S3). An analysis of the microbiome taxonomic composition in the tumors of different grades revealed differences in four genera. It is interesting to note that the relative abundance of the genus *Staphylococcus* was higher in low-grade tumors compared to high-grade ones (Table S3).

Though, we did not reveal significant difference in alpha-diversity between high grade and low grade tumors, and tumors at different stages, there was a tendency of a diversity increase in tumors of later stages and lower grades (*p* = 0.059 and *p* = 0.075, respectively).

### 3.3. Characterization of NSCLC Stroma

In this study, immunohistochemistry (IHC) was used to determine the possible correlation of the stromal cells phenotype and microbiome in NSCLC. An analysis of tumor stroma was done using CD68 for macrophages, iNOS for type 1 macrophages (M1), CD206 and CD163 for type 2 macrophages (M2), CD3 for T-cells, CD8 for cytotoxic T-cells and FoxP3 for Treg.

Analysis of iNOS expression revealed only very few iNOS-positive tumor associated macrophages (TAMs), however its expression was frequently found in tumor cells. We showed that increased expression of iNOS in tumor cells correlated with the histological type of tumor, namely, increased expression of this protein was observed in squamous cell carcinoma samples (*p* < 0.0001; Table 2). Additionally, squamous cell lung cancer was characterized by a higher content of CD68+ macrophages (*p* = 0.0343) and FOXP3+ regulatory T cells (*p* = 0.0014), compared with adenocarcinomas. Increased iNOS expression also correlated with tumor differentiation, namely, high iNOS expression was observed in highly differentiated tumors, which once again indirectly indicates that high differentiation is a favorable prognostic factor for NSCLC. It should also be noted that an increased content of both T cells in general (CD3+) and cytotoxic T cells (CD8+) are typical characteristics of the early stages of the disease (*p* = 0.0347 and *p* = 0.0343, respectively) and smaller tumors (*p* = 0.0179 and *p* = 0.0184, respectively; Table 2 and Table 3).

Next, we performed a quantitative analysis of bacteria in tumor tissue samples compared to adjacent normal lung tissue ones using real-time PCR. We showed that the total bacterial load in adjacent normal lung tissue was higher than in the tumor (two-sided Wilcoxon matched pairs signed rank test *p* = 0.0325 *).

**Figure 5:**
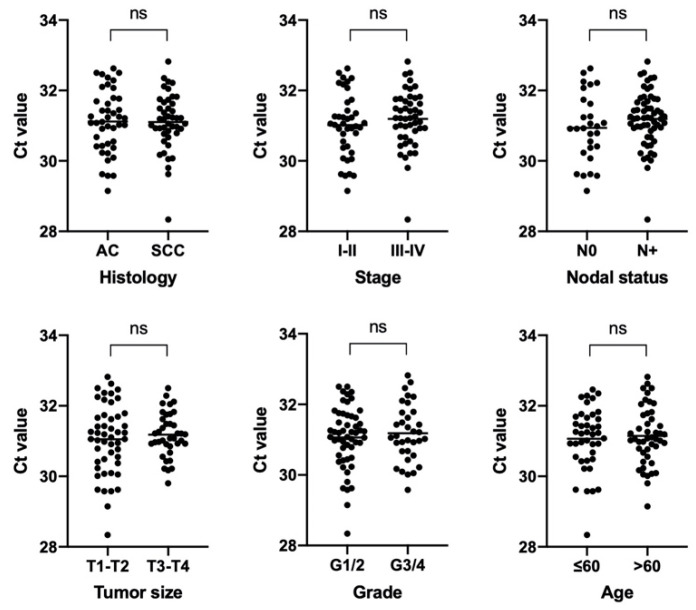
Real-time 16S PCR results as expressed by cycles to the cross threshold (Ct) for samples from patients with different clinicopathological characteristics. *p*-value evaluated with a U Mann–Whitney test.

Further, we checked whether the number of bacteria in the tumors differs depending on different clinical and histological features. The results are presented on Figure 5. There were no significant differences in the number of bacteria depending on clinical characteristics.

**Figure 6:**
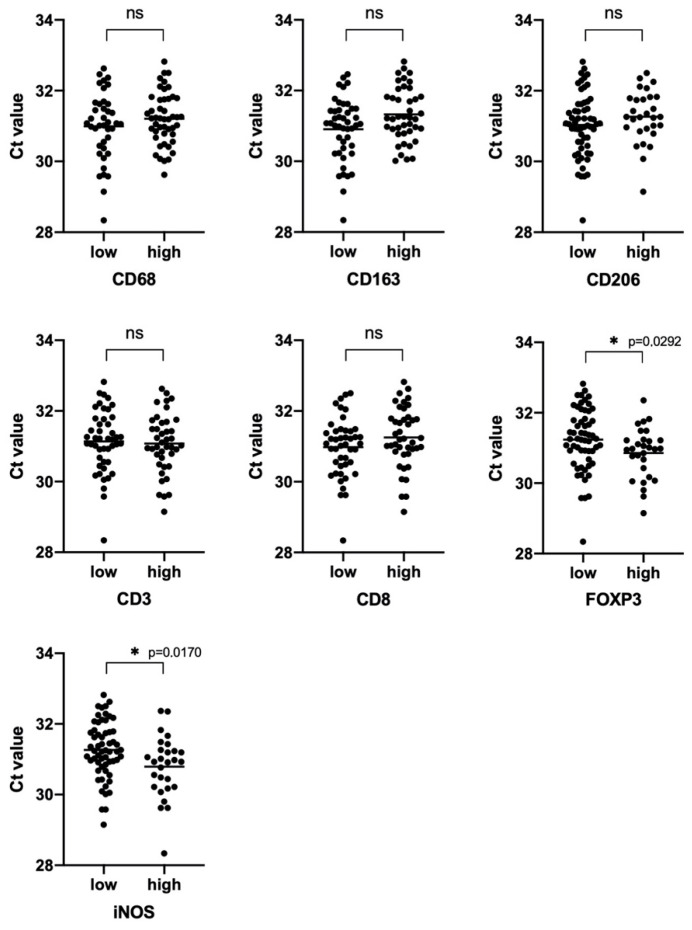
Real-time 16S PCR results as expressed by cycles to the cross threshold (Ct) for samples from patients with a different stroma phenotype. *p*-value evaluated with a U Mann–Whitney test. (∗- statistically significant).

We showed a significant difference in the total bacterial load of the samples with different levels of iNOS and FOXP3 expression (*p* = 0.0170 and *p* = 0.0292, respectively). In groups of samples characterized by high expression of these stromal markers, a higher bacterial load was observed (Figure 6).

### 3.4. Prognostic Significance of Studied Markers/Survival

It is known that some stromal tumor markers may have a prognostic value in NSCLC. We analyzed the survival of patients with NSCLC in groups with different levels of both iNOS and FOXP3 expression and depending on the total bacterial load. We also evaluated the combined contribution of these tumor features into overall patient survival.

**Figure 7:**
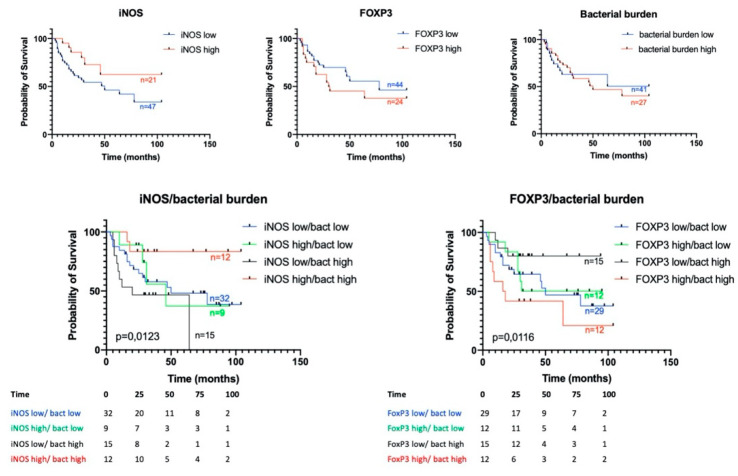
Kaplan–Meier curves of overall survival (OS) in NSCLC based on iNOS and FOXP3 expression and bacterial burden. For the curves the iNOS/bacterial burden and FoxP3/bacterial burden number at risk for 25, 50, 75 and 100 months are provided below the graphs.

We found that increased expression of iNOS by tumor cells seems to be a favorable prognostic factor, but this trend did not reach statistical significance (*p* = 0.0624). The total bacterial load, as well as the number of FOXP3 positive cells in the tumor, according to our data, are not prognostic markers and do not affect the overall survival of patients (Figure 7). Next, we analyzed the survival rate depending on the expression of the studied markers combined with the total bacterial load. We found that high iNOS expression accompanied by increased bacterial load is a marker of a good prognosis compared to the group of patients with high bacterial load and low iNOS expression (HR 0.1824 (0.05563–0.5983); *p* = 0.0123). It is worthy to note that in the group of cases with low bacterial load, the level of iNOS expression did not have a predictive capacity. At the same time, for the first time, we revealed that a high bacterial load of an immunosuppressed tumor (with a large number of FOXP3 + cells) is a marker of a poor prognosis in NSCLC compared with a group with a high bacterial load and low FOXP3 content (HR 4.651 (1.362–15.88); *p* = 0.0116; Figure 7). As it was found for iNOS, FOXP3 is only a predictive marker for a group of patients with a high bacterial load.

### 3.5. iNOS Features

**Figure 8:**
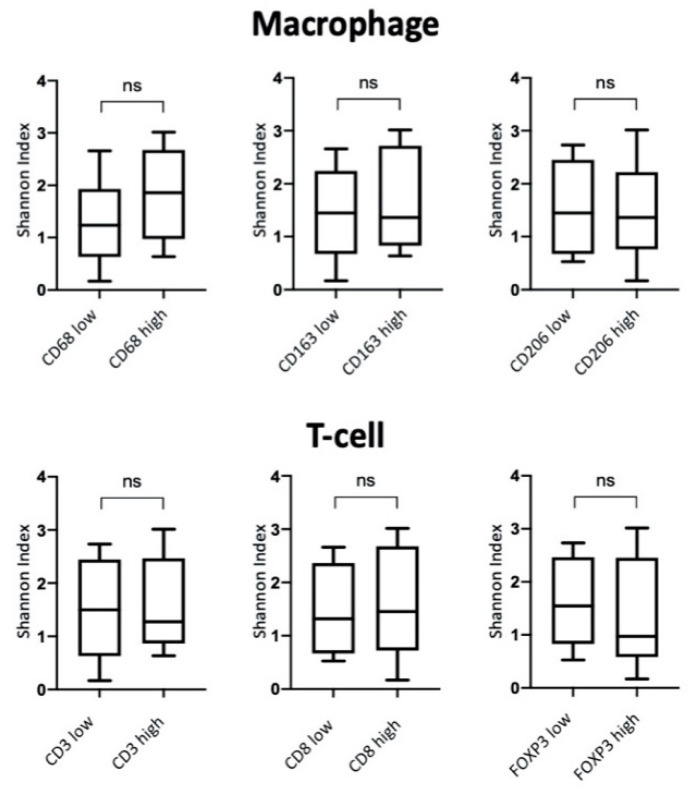
Taxonomic diversity between different groups depending on the phenotype of the inflammatory infiltrate.

To analyze the correlations between the alpha diversity of the bacterial communities and the phenotype of the tumor inflammatory infiltrate, 40 dominant bacterial genera with a relative abundance of at least 0.5% were taken, according to which the Shannon index was calculated. No statistically significant correlation with macrophage or T-cell markers was found (Figure 8).

**Figure 9:**
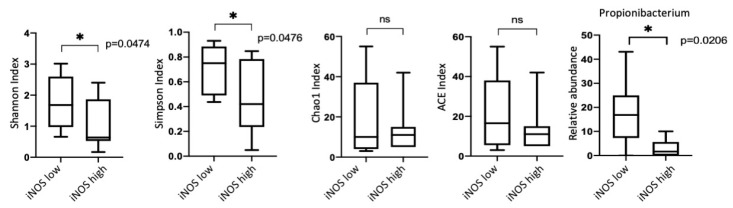
Taxonomic diversity between groups with different iNOS expression (∗-statistically significant).

The only statistically significant difference in microbiomes diversity was observed between the groups with different iNOS expression (Figure 9). We observed a significant decrease in the Shannon and Simpson indices in the group characterized by a higher iNOS expression. Taking into account that Shannon and Simpson indices are based on the number of taxa and their relative abundances, for groups with different levels of iNOS expression, we calculated additional indicators such as Chao1 and ACE indices, which characterize only the taxa number. We revealed that the studied groups did not differ in these indicators, which indicated that the differences in the Shannon and Simpson indices were due to only the relative abundance of the lung microbiome representatives. An increase in the Shannon and Simpson indices in the group with a low level of iNOS expression was found to be accompanied by an increase in the relative abundance of the only genus *Propionibacterium* (Figure 9). Additionally, the group with low iNOS expression showed a greater percentage of Gram-positive bacteria compared to Gram-negative bacteria (data not shown).

In general, for the first time we demonstrated that a high bacterial load of a tumor could be used as a bad or a good prognostic marker, depending on the phenotype of the tumor stroma and the state of local antitumor immunity.

## 4. Discussion

Commensal bacteria play an important role in maintaining the immune homeostasis of various organs and tissues, and disturbances in their balance can affect the susceptibility of the body to carcinogenesis or tumor progression. This study was aimed to investigate the lung tumor microbiome and its relation to the composition of its microenvironment and its prognostic significance.

The first part of the study was focused on characteristics of the lung microbiome in NSCLC tumors with different histopathologic features that help gaining insight into the possible role of a microbiome in lung cancer. The second part of the study was dedicated to the correlation of the NSCLC microbiome and the phenotype of tumor stroma and their joint impact on the disease outcome.

Theories of bacterial-mediated carcinogenesis have been proposed since the mid-20th century, when McCoy and Mason first suggested a link between *Enterococcus* and sigmoid carcinoma [25]. Sears and Pardoll formulated the “alpha bug” hypothesis, in which bacterial species *Bacteroides fragilis* plays a central pro-oncogenic role in producing enterotoxins, thereby contributing to colon cancer [26]. Subsequently, Tjalsma et al. in 2012 proposed a driver-passenger model, according to which driver-bacteria (e.g., *B. fragilis*) lead to multi-stage colorectal tumor carcinogenesis, including inflammation, increased cell proliferation and/or production of genotoxins [27]. Following the driver–passenger model, the “key hypothesis” of Hajishengallis et al. has been suggested. This hypothesis was based on key pathogens, which, even at low abundance, promote colonization by additional pathogens [28], followed by an inversion of the host response, resulting in an imbalance in the commensal microbiota and stimulation of the inflammatory response [29].

Lung cancer is a heterogeneous disease. Squamous cell carcinoma and adenocarcinoma are the two most common pathological types of lung cancer, which are characterized by different biological patterns, molecular biology and treatment strategies [30].

In this study, we have shown that the microbiome of adjacent normal lung tissue does not differ in taxonomic diversity at different taxonomic levels from tumor tissue, which is in good agreement with a recent study [31]. Representatives of the phyla *Actinobacteria, Proteobacteria, Firmicutes* and *Bacteroidetes* were predominant in the samples of tumors and adjacent normal tissues, which was also noted in other studies [32,33]. In contrast to our data on the lung tissue microbiome, the broncho-alveolar lavage microbiome in patients with lung cancer [33] was characterized by a higher relative abundance of the phylum *Fusobacteria* than the phylum *Actinobacteria*, which probably results from differences in the structure of the microbiomes on the surface of bronchi and within lung tissue, as well as from sampling procedures.

Despite the fact that squamous cell lung cancer is often associated with adverse effects of external factors (for example, smoking) and possible colonization of the lung by bacteria contained in tobacco in such patients [34], bacterial communities of lung tumors of various histological types did not differ in their taxonomic diversity (alpha diversity and Shannon index), which has been found also in previous research [6,32]. Interestingly, in our study, adenocarcinomas were characterized by a higher percentage of Gram-positive bacteria than Gram-negative ones, which may reflect the relationship between the microbiome composition and the histological type of tumor. Particularly, in patients with adenocarcinoma and squamous cell carcinoma 37 bacterial genera showed contrasting correlations with these subtypes of lung cancer [6].

There is a couple of publications describing the dynamics of quantitative and qualitative changes in the microbiome in the process of lung tumor progression, for instance, in the papers of Huang et al. [32] and Gomes et al. [6] there were no differences in alpha-diversity (Chao1, Shannon and Simpson indices) and beta-diversity (ordination of communities based on the Bray–Curtis distances). At the same time, the analysis of individual genera showed a significant decrease in the abundance of genus *Streptococcus* in adenocarcinomas with metastasis compared with ones without metastasis [32]. Besides, in the same study, in patients with lung squamous cell carcinoma, abundance of genera *Veillonella* and *Rothia* in tumors with metastasis was significantly higher than that in tumors without metastasis. In our study, the taxonomic composition of early and late stage lung tumors significantly differed, demonstrating differential distribution of 11 bacterial genera with constant alpha-diversity indices, which is in good agreement with the above findings, and indicates the need for further studies of the microbiome structure associated with lung cancer.

Further in our study, we carried out a quantitative analysis of the general bacterial load in the studied samples to assess its correlations with clinical characteristics. It is known from the literature that an increased bacterial load can be a poor prognostic marker for idiopathic pulmonary fibrosis [35], or contributes to the formation of lung tumors in vivo [36]. On the other hand, it is already known that the use of antibiotics prior to immunotherapy with checkpoint inhibitors significantly reduces the effectiveness of the antitumor treatment of NSCLC [37]. We showed that the content of bacteria in samples with different clinical characteristics does not differ, with the exception of groups of adjacent normal and tumor tissues. In the tumor tissue samples, a decrease in the total number of bacteria was observed, which indicates that the development of the tumor affects the normal local microbiota of the lung.

**Figure 10:**
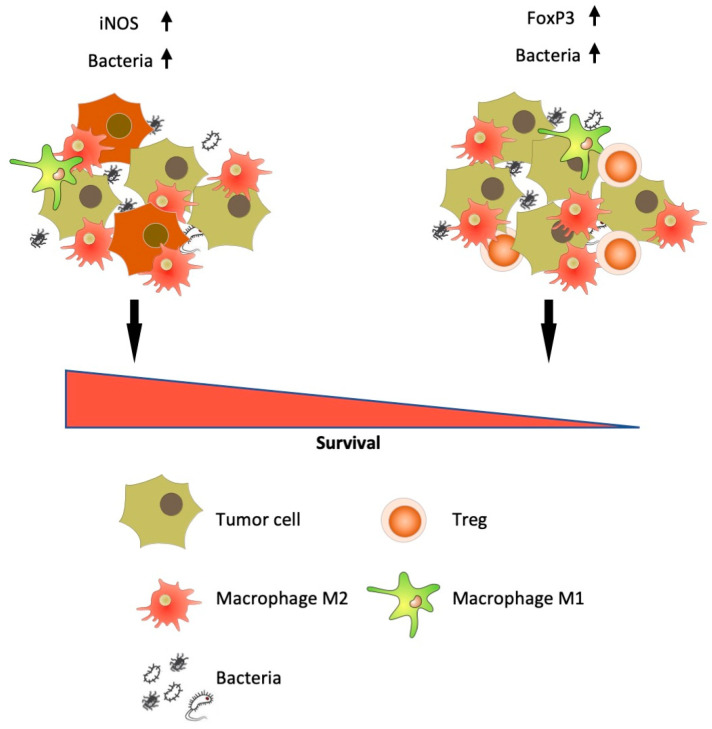
Bacterial load as an additional prognostic marker for NSCLC.

Next, we looked at the bacterial load in groups of tumors with different phenotypes of inflammatory infiltrate of tumor stroma. It is known that various stromal markers have their own individual, sometimes contradictory, prognostic significance. Thus, increased iNOS expression both in M1 macrophages and in NSCLC tumor cells can be a good prognostic factor [38,39,40]. We showed that increased expression of iNOS in tumor cells occurred in majority of the squamous cell lung cancer samples (*p* < 0.0001), and it is a favorable prognostic factor for NSCLC in general, but this indicator did not reach statistical significance. In general, the stroma of squamous cell carcinoma was characterized by a high content of CD68+ macrophages (*p* = 0.0343) and FOXP3+ regulatory T-cells (*p* = 0.0014), which indicates the distinctive properties of the microenvironment of this histological type of tumor. FOXP3 is more frequently considered an unfavorable prognostic marker for NSCLC [41]. We showed that high infiltration of FOXP3+ T-cells might be an unfavorable prognostic factor for squamous cell lung cancer. According to our data, for adenocarcinomas and NSCLC in general, FOXP3 cannot serve as a reliable prognostic criterion. Assessing the bacterial load in tumor samples with different stromal phenotypes showed that an increased bacterial content is typical for tumors with high iNOS (*p* = 0.0170) and FOXP3 (*p* = 0.0292) expression. Thus, we can assume that, on the one hand, a large number of bacteria in a tumor can trigger active inflammation processes (by increasing the expression of iNOS with subsequent production of NO). On the other hand, we found an increased number of bacteria in immunosuppressed tumors, containing strong infiltration of FOXP3+ cells. In this regard, at the next stage we are going to estimate, whether the phenotype of the tumor stroma in combination with the total bacterial load could be a prognostic marker of NSCLC (Figure 10).

We have shown that adding a score of the bacterial load to the phenotype of the tumor stroma drastically changed the prognostic value of the stromal markers. In the case of increased iNOS expression, a high bacterial load was a reliable favorable prognostic factor, while an increased bacterial load accompanied with a large number of FOXP3 cells was, on the contrary, a marker of a poor prognosis. This finding is in good agreement with the previously proposed concept that under the influence of incompletely established factors (possibly some “key” pathogens), an imbalance of the associated microbiome is formed in a tumor, and stimulation of the local inflammatory response of the body (in the case of preserved immunity) can occur [42]. That is why an increased content of bacteria in a tumor in combination with inflammatory markers can be a favorable prognostic factor.

## Figures and Tables

**Table 1:** Clinical characteristics of non-small cell lung cancer (NSCLC) patients.

Characteristic	Adenocarcinoma (AC; *n* = 44)	Squamous Cell Carcinoma (SSC; *n* = 45)	All (*n* = 89)
Age, mean ± SD	62.8 ± 10.5	61.8 ± 9.2	62.3 ± 9.9
Overall survival, mean ± SD	41.4 ± 27.7	36.5 ± 29.6	33.2 ± 19.2
Stage, *n* (%)	3 (7%)	2 (4%)	5 (6%)
I	21 (48%)	15 (33%)	36 (40%)
II	16 (36%)	26 (63%)	42 (47%)
II	4 (9%)	2 (4%)	6 (7%)
IV			
Differentiation, *n* (%)			
G1–G2	24 (55%)	31 (69%)	55 (62%)
G3–G4	20 (45%)	14 (31%)	34 (38%)
N, *n* (%)			
N = 0	19 (43%)	10 (22%)	29 (33%)
N³ 1	25 (57%)	35 (78%)	60 (67%)
T, *n* (%)			
T = 1–2	31 (70%)	19 (42%)	50 (56%)
T = 3–4	13 (30%)	26 (58%)	39 (44%)

**Table 2:** Analysis of clinicopathological characteristics of tumors and TAM markers.

	CD68			CD163			CD206			iNOS		
	High	Low	*p*	High	Low	*p*	High	Low	*p*	High	Low	*p*
Histology												
AC	18	26	0.0343 *	17	27	0.1387	14	30	0.8233	5	39	<0.0001 *
SCC	29	16		25	20		16	29		23	22	
Stage												
I–II	22	19	>0.999	21	20	0.5276	28	13	0.0578	12	29	0.8194
III–IV	25	23		21	27		23	25		16	32	
Nodal status												
N−	13	16	0.3666	13	16	0.8229	20	31	0.1703	6	22	0.1505
N+	34	26		29	31		9	29		23	38	
Tumor size												
T1–T2	27	23	0.8332	24	26	>0.9999	32	18	0.1957	13	37	0.2532
T3–T4	20	19		18	21		19	20		15	24	
Grade												
G1/2	27	28	0.3918	26	29	>0.9999	36	19	0.0769	23	32	0.0095 *
G3/4	20	14		16	18		15	19		5	29	
Age												
≤60	15	26	0.0059	13	28	0.0103 *	20	21	0.1969	17	24	0.0706
>60	32	16		29	19		31	17		11	37	

* Statistically significant.

**Table 3:** Clinicopathological characteristics and T-cell markers in NSCLC.

	CD3			CD8			FOXP3		
	High	Low	*p*	High	Low	*p*	High	Low	*p*
Histology									
AC	20	24	>0.9999	21	23	>0.9999	8	36	0.0014 *
SCC	21	24		22	23		23	22	
Stage									
I–II	24	17	0.0347 *	25	16	0.0343 *	13	28	>0.9999
III–IV	17	31		18	30		18	30	
Nodal status									
N−	16	25	0.2625	18	11	0.1124	8	21	0.6304
N+	13	35		25	35		23	37	
Tumor size									
T1-T2	29	21	0.0179 *	30	20	0.0184 *	16	34	0.6548
T3-T4	12	27		13	26		15	24	
Grade									
G1/2	25	30	>0.9999	27	28	>0.9999	21	34	0.6495
G3/4	16	18		16	18		10	24	
Age									
≤60	19	22	>0.9999	14	27	0.0191 *	10	31	0.0227 *
>60	22	26		29	19		21	27	

* Statistically significant.

## Supplementary Materials

The following are available online at https://www.mdpi.com/2227-9059/8/9/349/s1. Five tables are provided as supplementary material in a single file.
