# Supplementary material for: Lung Microbiome Differentially Impacts Survival of Patients with Non-Small Cell Lung Cancer Depending on Tumor Stroma Phenotype
Source: Biomedicines. 2020 Sep 13;8(9):349. doi: 10.3390/biomedicines8090349 (PMC7554830; doi:10.3390/biomedicines8090349)
Supplement: Supplementary file 1 [file biomedicines-08-00349-s001.pdf]

Supplementary data

Supplementary table 1. Taxonomic composition of the microbial community of lung tissues on the level of phylum.

Phylum	**N**	**T**	**P value**
Actinobacteria	37,71	36,06	0,73
Proteobacteria	28,94	33,61	0,36
Firmicutes	15,33	14,97	0,33
Cyanobacteria_Chloroplast	5,92	8,31	0,93
Bacteroidetes	4,46	5,60	0,15
Parcubacteria	0,04	0,65	0,75
Others	1,60	0,50	NA
Fusobacteria	0,12	0,21	0,69
unclassified_Bacteria	2,34	0,06	0,06
Candidatus_Saccharibacteria	0,97	0,01	0,50
Tenericutes	2,52	0,00	0,50

Supplementary table 2. Taxonomic composition of the microbial community of lung tissues on the level of genera.

	**N**	**T**	**P value**
Propionibacterium	19,66	14,18	0,11
Corynebacterium	11,40	10,95	0,28
Sphingomonas	1,59	10,58	0,08
Cyanobacteria unclassified	6,29	9,20	0,84
Staphylococcus	10,15	8,96	0,18
Ralstonia	3,58	6,69	0,61
Haemophilus	0,22	2,33	0,79
Shewanella	0,02	2,16	0,75
Streptococcus	1,13	2,06	0,40
Porphyromonas	0,04	1,82	0,44
Melissococcus	0,00	1,77	0,50
Burkholderia	0,74	1,75	0,36
Aquabacterium	0,58	1,61	0,16
Terrimonas	0,00	1,42	>0,9999
Parvimonas	0,03	1,41	0,63
Rothia	0,62	1,38	0,97
Brevibacterium	0,48	1,23	0,81
Roseomonas	1,23	1,22	0,11
Mycobacterium	0,01	1,16	>0,9999
Kosakonia	0,00	1,13	0,50
**Acinetobacter***	**5,33**	**1,06**	**0,05**
Enterobacter	0,01	0,91	0,50
Paracoccus	1,69	0,90	0,10
Escherichia/Shigella	0,14	0,89	0,13
Serinicoccus	0,02	0,88	0,50
Williamsia	0,00	0,87	>0,9999
Microbacterium	0,03	0,87	>0,9999
Pseudomonas	0,64	0,83	0,70
**Halomonas***	**1,64**	**0,72**	**0,02**
Neisseria	1,67	0,65	0,68
Micrococcus	0,83	0,61	0,41
Comamonas	3,46	0,56	0,81
Anaerococcus	0,33	0,54	0,12
Massilia	0,15	0,48	0,47
Kocuria	0,86	0,48	0,58
Nesterenkonia	0,33	0,41	>0,9999
Variovorax	0,04	0,40	0,41
Okibacterium	1,65	0,38	0,09
Enhydrobacter	0,73	0,37	0,61
Arthrobacter	0,17	0,36	0,91
Finegoldia	0,20	0,33	0,90

Altererythrobacter	0,01	0,29	0,25
Actinomyces	0,40	0,29	0,53
Pseudoalteromonas	0,00	0,26	>0,9999
Lactobacillus	0,08	0,26	0,69
Haematobacter	1,31	0,23	0,32
Fusobacterium	0,05	0,22	0,16
Rhodococcus	0,12	0,16	>0,9999
Janibacter	0,13	0,15	0,84
Dermacoccus	0,66	0,13	0,16
Brevundimonas	0,62	0,11	0,31
Prevotella	1,06	0,09	0,41
Veillonella	0,69	0,09	>0,9999
Nocardioides	0,19	0,08	0,09
Flavobacterium	0,17	0,08	0,31
Granulicatella	0,15	0,06	0,31
Rhizobium	0,70	0,05	0,56
Bacillus	1,72	0,04	0,81
Pseudoclavibacter	0,88	0,04	0,20
Bacteroidetes	0,56	0,03	>0,9999
Empedobacter	0,26	0,02	0,63
Bosea	0,62	0,02	0,16
Saccharibacteria_genera_incertae_sedis	1,08	0,02	0,50
Methylobacterium	2,14	0,01	0,41
**Chryseobacterium***	**2,36**	**0,01**	**0,03**
Exiguobacterium	1,21	0,00	>0,9999
Luteimonas	0,14	0,00	0,13
Mycoplasma	2,80	0,00	0,50
Carnobacterium	0,14	0,00	>0,9999
Saccharopolyspora	1,44	0,00	0,50
Others	0,63	0,11	NA

Supplementary table 3. Taxonomic composition of the microbial communities in lung tumors of different histological types at the genus level

Genus	AC	SCC	p
Propionibacterium	15,04	11,55	0,64
Corynebacterium	5,87	12,87	0,17
Sphingomonas	5,23	12,95	0,41
Cyanobacteria unclassified	12,02	4,98	0,49
Staphylococcus	10,03	4,42	0,87
Ralstonia	2,64	8,97	0,27
Haemophilus	3,48	0,13	0,30
Shewanella	0,06	3,81	0,74
Streptococcus	1,15	2,37	0,17
Porphyromonas	0,00	3,28	0,08
Melissococcus	2,73	0,00	0,48
Burkholderia	2,30	0,47	0,44
Aquabacterium	2,31	0,20	0,45
Terrimonas	0,00	2,56	0,46
Parvimonas	0,01	2,54	0,36
Rothia	1,75	0,45	>0,9999
Brevibacterium	1,90	0,00	0,22
Roseomonas	1,87	0,00	0,48
Mycobacterium	0,00	2,08	0,46
Kosakonia	1,74	0,00	0,48
Acinetobacter	0,96	6,64	0,84
Enterobacter	1,27	0,16	>0,9999
Paracoccus	0,40	1,14	0,51
Escherichia/Shigella	0,27	1,28	0,63
Serinicoccus	1,22	0,16	0,58
Williamsia	1,34	0,00	>0,9999
Microbacterium	0,52	0,96	0,74
Pseudomonas	4,42	1,08	0,97
Halomonas	0,87	0,29	0,72
Neisseria	0,07	1,09	0,48
Micrococcus	0,44	0,58	0,92
Comamonas	0,00	1,00	0,80
Anaerococcus	0,77	0,07	0,45
Kocuria	0,46	0,32	0,84
Okibacterium	0,18	0,46	>0,9999
Enhydrobacter	0,33	0,28	0,87
Haematobacter	0,18	0,22	>0,9999
Dermacoccus	0,09	0,14	0,87

Brevundimonas	0,03	0,15	0,45
Prevotella	0,05	0,10	0,60
Veillonella	0,05	0,11	0,87

Таблица 4. Таксономический состав опухолей легкого в зависимости от стадии заболевания

**Genus**	Stage I-II	Stage III-IV	
Propionibacterium	14,95	12,72	0,75
Cyanobacteria unclassified	12,35	6,29	0,74
Corynebacterium	9,19	9,61	**0,02**
Staphylococcus	7,14	8,30	0,11
Acinetobacter	6,28	1,85	0,29
Sphingomonas	5,68	12,04	**0,03**
Pseudomonas	5,09	1,56	**0,03**
Haemophilus	4,36	0,44	0,81
Porphyromonas	3,57	0,34	0,34
Burkholderia	3,32	0,45	**0,03**
Aquabacterium	3,24	0,29	**0,03**
Roseomonas	2,54	0,29	0,20
Kosakonia	2,38	0,29	0,20
Brevibacterium	2,38	0,45	>0,9999
Williamsia	1,92	0,29	0,46
Enterobacter	1,83	0,43	0,74
Ralstonia	1,41	9,71	0,07
Anaerococcus	1,15	0,44	0,60
Streptococcus	1,06	2,87	**0,02**
Paracoccus	0,95	1,16	0,10
Okibacterium	0,74	0,54	0,97
Neisseria	0,66	1,03	**0,04**
Micrococcus	0,63	0,99	0,10
Haematobacter	0,63	0,41	0,54
Enhydrobacter	0,47	0,76	0,07
Halomonas	0,43	1,34	**0,04**
Kocuria	0,41	0,98	**0,04**
Escherichia/Shigella	0,41	1,61	0,06
Dermacoccus	0,38	0,47	0,38
Brevundimonas	0,37	0,44	0,29
Comamonas	0,35	1,15	0,31
Prevotella	0,35	0,43	0,38
Shewanella	0,35	3,62	0,48
Melissococcus	0,35	3,02	0,48
Terrimonas	0,35	2,48	>0,9999
Parvimonas	0,35	2,47	**0,04**
Rothia	0,35	2,43	**0,02**
Mycobacterium	0,35	2,08	>0,9999
Serinicoccus	0,35	1,65	0,22
Microbacterium	0,35	1,63	0,48
Veillonella	0,35	0,43	0,10

Таблица 5. Таксономический состав опухолей легкого в зависимости от дифференцировки опухоли

**Genus**	G1/G2	G3/G4	P value
Sphingomonas	15,40	1,77	0,60
Propionibacterium	11,99	15,81	0,41
Corynebacterium	9,08	9,82	0,33
Ralstonia	8,89	2,37	0,38
Acinetobacter	6,08	1,36	0,93
Staphylococcus	5,46	10,45	**0,03**
Pseudomonas	4,69	1,45	0,55
Cyanobacteria unclassified	3,69	15,38	**0,01**
Haemophilus	2,91	1,48	0,77
Melissococcus	2,91	0,48	>0,9999
Porphyromonas	2,86	0,64	0,58
Streptococcus	2,52	1,47	0,82
Aquabacterium	2,41	0,77	0,53
Terrimonas	2,37	0,48	>0,9999
Parvimonas	2,35	0,49	0,78
Roseomonas	2,05	0,48	>0,9999
Brevibacterium	1,92	0,67	0,58
Rothia	1,64	1,27	0,77
Williamsia	1,52	0,48	>0,9999
Escherichia/Shigella	1,36	0,70	0,25
Burkholderia	1,32	2,31	0,77
Comamonas	1,04	0,48	0,53
Microbacterium	0,70	1,44	0,74
Halomonas	0,50	1,41	0,18
Micrococcus	0,42	1,30	0,35
Kocuria	0,37	1,12	0,21
Enterobacter	0,32	1,96	0,74
Prevotella	0,30	0,51	0,45
Veillonella	0,29	0,51	0,53
Neisseria	0,29	1,52	0,24
Okibacterium	0,28	1,04	0,43
Haematobacter	0,23	0,84	0,06
Enhydrobacter	0,23	1,09	0,08
Dermacoccus	0,22	0,67	0,20
Paracoccus	0,21	2,05	**0,01**
Brevundimonas	0,19	0,67	0,06
Kosakonia	0,18	2,51	0,74
Shewanella	0,18	4,36	0,20
Mycobacterium	0,18	2,56	0,46
Serinicoccus	0,18	2,07	0,08
Anaerococcus	0,18	1,44	**0,03**

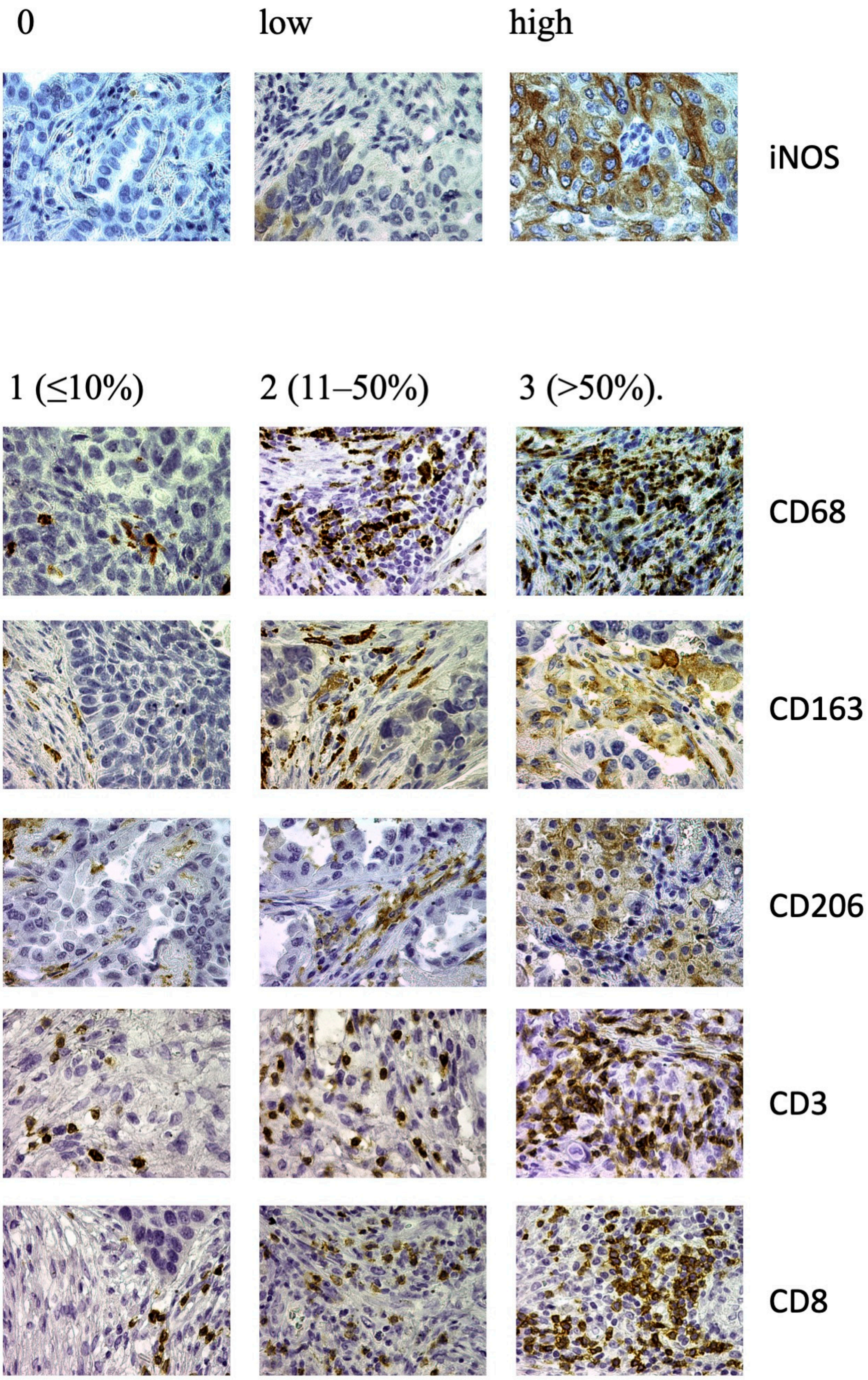

Supplementary figure 1. Examples of immunohistochemical staining with iNOS and various macrophage and T-cell markers.

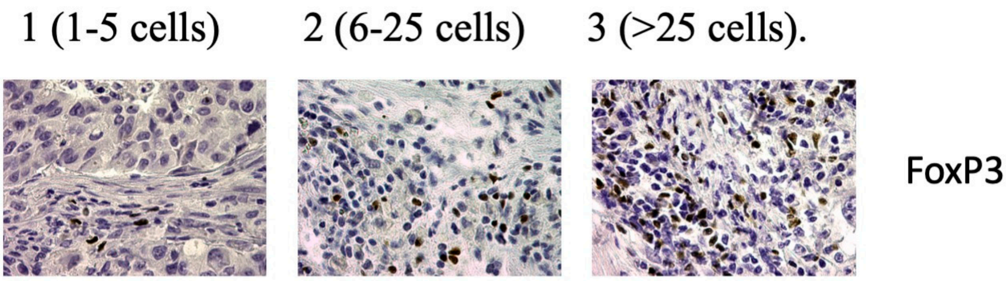

Supplementary figure 2. Examples of immunohistochemical staining with FoxP3.
